# Cost-Effectiveness of Routine Histopathological Analysis of Doughnuts after Colorectal Surgery Three-Year Single-Centre Experience

**DOI:** 10.1155/2024/9837336

**Published:** 2024-08-19

**Authors:** Masood Ur Rehman, Reem Moussa, Cindy Siaw Lin, Naeem Ahmed, Abdul Rehman, Kamran Malik, Jamil Ahmed

**Affiliations:** Department of Colorectal Surgery Northampton General Hospital, Northamptonshire, UK

## Abstract

**Aim:**

This study aimed to assess the impact of routine histological examination of stapled colorectal anastomotic doughnuts in patients undergoing rectal cancer surgery (RCS). Justification of biopsy examination could form part of the strategies of NHS net zero practice with effort to reduce wastage and carbon footprint.

**Method:**

A data analysis of all patients undergoing RCS during 2019–2021 at our institute was performed. We also analysed the cost of preparing and reviewing histology slides.

**Results:**

52 patients underwent anterior resection during the aforementioned period. Doughnuts were sent in 37 (71%) patients. 23 (62%) patients were male, and 14 (38%) were female. The median age at diagnosis was 68 (range 54–84) years. All resected specimens were adenocarcinomas. Of the 37 patients, 18 (49%) underwent low anterior resection and 19 (51%) underwent high anterior resection. Proximal doughnuts were sent in 26 (70%) patients, whereas distal doughnuts were sent in all cases. Mean distal microscopic resection margin from tumour was 22 mm (range 6–45 mm). Each doughnut required 3 slides, each costing £50 and requiring 82 minutes to fix and read. This incurred a cost of £13,650 and required 19,656 hours of preparation time. All of the doughnuts as well as resection margins were negative for malignancy.

**Conclusion:**

Routine histopathological examination of doughnuts is time and cost-intensive however provides little or no clinical value (particularly analysis of the proximal doughnut). Distal doughnuts should only be sent for histological examination in exceptional circumstances.

## 1. Introduction

Colorectal cancer is one of the most common cancers comprising about 1.4 million new cases worldwide [1]. There are around 42,900 new bowel cancers diagnosed in the UK every year making it the 4^th^ most common cancer in the UK [2]. Surgery is the main course of treatment to cure colorectal cancer. The circular stapling gun is routinely used to perform colorectal anastomosis after colorectal resection. It reduces the need for hand-sewn anastomosis due to difficulty in accessing in deep pelvis [3, 4].

In 1979, the first stapling gun was described to facilitate low colorectal end-to-end anastomosis (CEEA) [5]. The double stapling technique was described by Griffin and Knight in which the distal rectal stump is closed by a linear stapler, and subsequently, the anastomosis is carried out by a circular stapler [6]. The transection of rectum distal to the tumour is performed with the linear stapler. A circular staple gun of appropriate size is selected for colorectal anastomosis. The anvil of the circular stapler is secured at the distal end of the mobilised colonic conduit with purse-string suture. The gun is inserted through the rectum to securely dock the anvil onto the end of the stapler gun. Following approximation, the stapler gun inserted from the rectum is fired to complete the anastomosis. This gun is very beneficial in performing the anastomosis safely and efficiently particularly in the lower rectum [3, 4].

The two complete rings also known as doughnuts, one from the colonic side (proximal) and the other from the rectal stump side (distal), are achieved at the completion of anastomosis. A careful evaluation of these doughnuts is important because their intactness and thickness reflect intact anastomosis in addition to the benefits of its microscopic evaluation in oncological outcomes. However, identification of cancer cells in the doughnuts and its impact on further oncological treatment remains unclear [7, 8]. Additionally, stapling devices may lead to crushing and rupture of the tissues which can impair histological reviews leading to low detection rates of cancer and thus little clinical implication [9].

It is usually not necessary to perform microscopic examinations of doughnuts from stapling devices if the tumour is greater than 30 mm from the longitudinal margin of the main specimen [10]. However, there are no clear clinical guidelines from any leading colorectal societies regarding clinical implication of microscopic examination of doughnuts [6–8]. Some studies argue that this practice is time consuming and not cost-effective [9]. Additional biopsy processing could also have significant environmental impact not only through the direct emission of greenhouse gases but also indirect emissions from energy consumption in laboratories [9].

A recent systematic review of 8 studies including 1,754 patients revealed only less than 1% positive results for cancer in distal doughnuts and no overall change in the clinical outcomes [2]. We conducted this retrospective study in our department to assess the cost-effectiveness and clinical benefit of routine histopathological examination of doughnuts after anterior resection in colorectal cancer surgery. With the increasing awareness of surgical carbon footprinting and NHS net zero effort, the ultimate aim is to achieve consensus regarding the necessity of this routine practice which carries impact on healthcare sustainability.

## 2. Method

A retrospective data analysis of all patients who underwent elective anterior resection during 2019–2021 was performed. All patients who underwent colorectal anastomosis using circular stapler were included, including those who received neoadjuvant therapy. We analysed patients' demographics and surgical procedure along with assessment of histopathological reports.

All histopathological reports were issued by pathologists with a special interest in lower gastrointestinal tract cancers and were compliant with the Royal College of Pathologists' guidelines for colorectal cancer.

Patients who underwent abdominoperineal resection and permanent colostomy were excluded due to the unavailability of doughnuts. Variables recorded included age, gender, circumferential resection margin (CRM), clinical T stage, the involvement of proximal and distal margins, the involvement of proximal and distal doughnuts, length of proximal and distal margins, and length of proximal and distal doughnuts. Margins were deemed positive if the tumour was found within 1 mm from the resection margin.

## 3. Results

### 3.1. Clinical Benefit

A total of 91 patients who underwent colorectal resections were included in the study. Eight were excluded due to incomplete data. Only 52 patients had anterior resection (AR) with colorectal anastomosis during the aforementioned period. Doughnuts were sent for histopathological analysis in 37 (71%) patients. 23 (62%) patients were male, and 14 (38%) were female (Table 1). The median age at diagnosis was 68 (range 54–84) years. All resected specimens were reported as adenocarcinoma.

### 3.2. TNM Staging and Distance from Resection Margin

The most common tumour stage was T3 (37.84%) followed closely by T2 (32.43%) (Table 2). The shortest distance from the distal resection margin was 6 mm, and the mean distal microscopic resection margin from tumour was 22 mm (range 6–45 mm) (Table 3). All proximal margins were negative for cancer involvement. There was one R1 resection as CRM was positive on the right lateral side.

Among the 37 patients, distal doughnuts were sent in all (100%) of the patients, whereas both proximal and distal doughnuts were sent in 26 (70%) cases. None of the doughnuts were positive for cancer (Table 3).

#### 3.2.1. Cost Analysis

Each slide at the Northampton General Hospital costs 50 GBP. Three slides are usually prepared for each doughnut, 72 hours and 10 minutes, respectively, are required to fix and subsequently read each slide. Ninety-one cases were carried out in total, costing £13,650 and requiring 19,656 hours of preparation time.

## 4. Discussion

Our data have shown that there is no significant benefit of routine evaluation of end-to-end anastomosis doughnuts in this cohort of patients. None of the doughnuts were deemed positive for cancer cells on microscopic examination, and this has been shown in multiple recent studies [11, 12]. Furthermore, the use of these doughnuts has been shown to have a significant impact on cost, human resources, and carbon footprint.

Laparoscopic surgery along with CEEA has significantly increased the chances of sparing the anal sphincter in many patients, meaning that the need for a permanent colostomy will have reduced and subsequently the quality of life is preserved in many patients [13]. It is common practice that the proximal and distal doughnuts' yield which theoretically reflects the true margins are sent for microscopic examination [10]. Many centres recommend the histopathological examination of these doughnuts [14]. It is important to note that the margins obtained from doughnuts are not always representative of the true margin, as for instance they cannot represent the true distal margins if location of the tumour is on the anterior wall of the colon or rectum and the gun is fired posteriorly to the staple line and vice versa. As there is no defined standard for the preferred stapler needle penetration through the distal end of bowel (it can penetrate anteriorly, posteriorly, through, and to the right or left of the circumference), this can confound the definition of the true proximal and distal margin of doughnuts, thereby producing unreliable results.

Following colorectal surgery, the length of margins to take has remained controversial. Recent studies have shown that even margins of less than 5 mm do not affect oncological outcomes [11, 12]. Moreover, the intention to spare the sphincter may result in an increase in the risk of positive margins. Hence, the significance of microscopic examination of the anastomotic doughnut has remained unclear. Some studies recommend histopathological examination of only distal margins of less than 2 cm, while others do not [7, 15, 16].

It is also important to note that length of the distal margin is still debatable, despite the evidence that the longitudinal spread is very rare in cancer size greater than 1 cm [17]. A study by Hughes and Jenevin assessed 42 colorectal cancers and found evidence of intramural spread in only 4.7% of cases [18], whereas spread of less than 2 cm was found in most studies except for one case shown by Sidoni [19]. In another study, longitudinal spread of at least 1 cm is seen in a rectal cancer patient [20]. Therefore, 2-cm distal length margin is considered adequate for the negative margin, and thus, the operating surgeon should ensure that a negative margin of 2 cm is reached for low rectal cancers, and greater margins are achieved in high rectal tumours [7, 15, 16, 21]. Hence, it is important to specimen the doughnut in theatre following resections and only send the distal doughnut if the distal margin is confirmed to be positive.

On the other hand, microscopic evaluation of doughnuts may play an important role in aggressive cancer types such as small cell carcinoma, undifferentiated cancers, pure signet cell cancers, or cancers with extensive lymph or vascular invasion. Nevertheless, there are certain situations where a microscopic analysis is warranted [17, 22–25]. Some studies recommend performing the microscopic examination of the distal doughnut in which the distal margin is less than 2 cm. Our study however did not show any doughnut positivity.

The incidence of carcinoma found in doughnuts after resection is 0.5–0.8% [7, 15, 16, 21]. In our study, none of the specimens were marked positive and none of the margins were positive for cancer. Therefore, our results suggest that the use of doughnuts does not have any impact on further treatment plans. Doughnuts do not need to be examined except in rectal adenocarcinoma with neoadjuvant therapy given prior to the surgery as in these cases there may not be any mucosal evidence of malignancy and microscopic examination will be needed to assure complete excision [10].

Carbon footprinting is a “measure of carbon dioxide emissions (C02e) into the atmosphere as a result of the activities of a particular individual, organisation, or community” [26]. The carbon footprint of healthcare systems around the world have been well studied, with research to suggest that the healthcare sector is responsible for up to 5% of global greenhouse gas emissions [27]. In 2019, NHS England was responsible for 25 megatonnes (Mt) of C02 emissions. 62% [15.6 Mt C02e] of these emissions were produced as a result of the supply chain, of which medical equipment and pharmaceuticals and chemicals (such as tissue processing equipment) made up a substantial proportion (51%; [8.1 Mt C02e]) [28]. The histopathological examination of colorectal cancer specimens requires the utilization of a variety of reagents and equipment to ensure the highest quality pathology reporting. Gastrointestinal biopsies play a significant role in such greenhouse gas emissions; in fact, one study found that up to 0.79 kg of C02e are produced by each specimen sent from a GI biopsy, with most emissions emerging from tissue processing (36%) and the production of single use jars (16%). In a 20 million person population, the greenhouse gas emissions produced are equivalent to the annual emissions of over 1000 cars [29]. Given the prevalence of colorectal cancer in UK, routine histopathological examination of doughnuts could have significant contribution to healthcare associated emissions. Hence, the establishment of criteria for requesting histopathological examination of doughnut in rectal cancer resection is a noteworthy strategy to reduce surgical carbon footprint. It is therefore paramount to evaluate the necessity of sending two specimens for histopathological evaluation following each end-to-end colorectal anastomosis, as a reduction in unnecessary samples will ultimately lead to a reduction in C02 emissions, which is in keeping with the current strategy to achieve the NHS Long-Term Plan and reach Net Zero targets [30, 31]. Whilst data regarding the carbon footprint of slides produced at our institution were not available, the aforementioned research highlights the importance of economic use of doughnut examination order to avoiding the harmful environmental consequences, particularly as our paper has shown that distal margins were negative and did not contribute to management or health outcomes.

The time required to prepare histopathological slides and cost-effectiveness varies across the various health systems and institutes. In some centres in the United States, histopathological analysis of one doughnut sample can cost up to $643 [32]. In the United States, roughly 150,000 colorectal carcinoma cases are performed per year [33], and with results from histological analysis of doughnuts offering little to no value in the management process, their use should be reconsidered. Further studies have demonstrated the lack of cost-effectiveness of colorectal doughnut anastomosis samples. One study by Dixon et al. [9] discussed that preparation for a single doughnut at a centre in the UK requires 36 hours at a cost of £8 per doughnut. Given the sheer number of doughnut samples that are sent, particularly both proximal and distal, it is difficult to justify the analysis of each doughnut given the low detection rates for cancer. This is further supported by Haq et al. who argue that not sending every doughnut for histology can save up to $5,000 annually [34]. At our institute, 72 hours are required to prepare each slide, at a cost of £50. Over a three-year period, we have not found any doughnuts to be positive for cancer, and therefore, the management of these patients has not been altered in any way by analysing the doughnuts. We therefore advise against sending every doughnut for histopathological evaluation in order to save time and effort of colleagues running the samples, cost, and carbon footprint.

## 5. Recommendations

We must establish our local institutional based guidelines for histopathological analysis of the doughnuts which helps in saving a significant amount of time and cost. Only doughnuts which contain positive margins of the primary specific should be examined. It is important to remember that doughnuts are not truly representative of cancer margins. This strategy has also been endorsed by the American College of Pathologists [8].

## 6. Conclusion

As routine histopathological examination of end-to-end anastomotic doughnuts does not affect outcomes and management of the disease, careful selection of specimen should be performed. We recommend that doughnuts should be sent with the primary specimen and only be examined if the specimen margins are positive. Our study also suggests that only distal doughnuts should be sent in those in which there is a higher suspicion of possibility of a positive margin. The wide implementation of this practice could optimise laboratory resources' utilization contributing to the effort of promoting global sustainability.

## Figures and Tables

**Table 1:** Showing ratio of male to female participants and tumour site.

Total = 37	*N*	Percentage (%)
Gender		
Female	14	37.84
Male	23	62.16
Tumour site		
Low rectal	2	5.41
Midrectum	18	48.65
Upper rectal	17	45.95

**Table 2:** Showing tumour stages.

	*N*	Percentage (%)
Tumour stage		
1	10	27.03
2	12	32.43
3	14	37.84
4	1	2.70
Nodal stage		
N		
0	26	70.27
1	9	24.32
2	2	5.41
R		
0	36	97.30
1	1	2.70

**Table 3:** Showing percentage of samples sent for analysis and subsequent proportion samples positive for cancer, as well as distance from resection margin.

	Specimen sent	Percentage (%)	Positive for cancer	Distance from resection margin
Proximal and distal doughnuts	26	70.27	None	N/A
Distal doughnuts only	37	100	None	6−45 mm (mean 22 mm)

## Data Availability

The data that support the findings of this study are available from the corresponding author upon reasonable request.

## References

[1] Ferlay J., Soerjomataram I., Ervik M., Dikshit R., Eser S., Mathers C. (2012). IARC cancer base, 10. France: international agency for research on cancer. *Cancer Incidence and Mortality Worldwide*.

[2] Wlodarczyk J., Gaur K., Mertz K. (2022). Do or doughnut: a systematic review and pooled analysis on the utility of pathological evaluation of the anastomotic doughnut in oncological colorectal operation. *Colorectal Disease*.

[3] Idrees R., Fatima S., Abdul-Ghafar J., Raheem A., Ahmad Z. (2018). Cancer prevalence in Pakistan: meta-analysis of various published studies to determine variation in cancer figures resulting from marked population heterogeneity in different parts of the country. *World Journal of Surgical Oncology*.

[4] Ho Y.-H., Ashour M. A. T. (2010). Techniques for colorectal anastomosis. *World Journal of Gastroenterology*.

[5] Moran B. (1996). Stapling instruments for intestinal anastomosis in colorectal surgery. *British Journal of Surgery*.

[6] Knight C. D., Griffen F. D. (1980). An improved technique for low anterior resection of the rectum using the EEA stapler. *Surgery*.

[7] Speake W. (2003). Should “doughnut” histology be routinely performed following anterior resection for rectal cancer?. *The Annals of the Royal College of Surgeons of England*.

[9] Dixon S., Barrow H. (2020). Donoughts bother, Histopathological examination of anastomotic donoughts following colorectal anastomosis does not change patient management. *Surgical and Experimental Pathology*.

[10] Morlote D. M., Alexis J. B. (2016). Is the routine microscopic examination of proximal and distal resection margins in colorectal cancer surgery justified?. *Annals of Diagnostic Pathology*.

[11] Fitzgerald T. L., Brinkley J., Zervos E. E. (2011). Pushing the envelope beyond a centimetre in rectal cancer: oncologic implications of close, but negative margins. *Journal of the American College of Surgeons*.

[12] Kiran R. P., Lian L., Lavery I. C. (2011). Does a sub centimetre distal resection margin adversely influence oncologic outcomes in patients with rectal cancer undergoing restorative proctectomy?. *Diseases of the Colon & Rectum*.

[13] Luukkonen P., Järvinen H. (1993). Stapled vs hand-sutured ileoanal anastomosis in restorative proctocolectomy: a prospective, randomized study. *Archives of Surgery*.

[14] Smith A. J., Driman D. K., Spithoff K. (2010). Guideline for optimization of colorectal cancer surgery and pathology. *Journal of Surgical Oncology*.

[15] Cross S., Bull A. (1989). Is there any justification for the routine examination of bowel resection margins in colorectal adenocarcinoma?. *Journal of Clinical Pathology*.

[16] Pullyblank A., Kirwan C., Rigby H., Dixon A. (2001). Is routine histological reporting of doughnuts justified after anterior resection for colorectal cancer?. *Colorectal Disease*.

[17] Williams N., Dixon M., Johnston D. (1983). Reappraisal of the 5-centimetre rule of distal excision for carcinoma of the rectum: a study of distal intramural spread and of patients’ survival. *British Journal of Surgery*.

[18] Hughes T. G., Jenevein E. P. (1983). Intramural spread of colon carcinoma: a pathologic study. *The American Journal of Surgery*.

[19] Sidoni A., Bufalari A., Alberti P. F. (1991). Distal intramural spread in colorectal cancer: a reappraisal of the extent of distal clearance in fifty cases. *Tumori Journal*.

[20] Shimada Y., Takii Y., Maruyama S., Ohta T. (2011). Intramural and mesorectal distal spread detected by whole-mount sections in the determination of optimal distal resection margin in patients undergoing surgery for rectosigmoid or rectal cancer without preoperative therapy. *Diseases of the Colon & Rectum*.

[21] Morgan A., Dawson P., Smith J. J. (2006). Histological examination of circular stapled “doughnuts”: questionable routine practice?. *Surgeon*.

[22] Loughrey M. B., Quirke P., Shepherd N. A. (2014). Standards and datasets for reporting cancers dataset for colorectal cancer histopathology reports. *The Royal College of Pathologist*.

[23] Quirke P., Dixon M., Durdey P., Williams N. (1986). Local recurrence of rectal adenocarcinoma due to inadequate surgical resection: histopathological study of lateral tumour spread and surgical excision. *The Lancet*.

[24] Adam I., Martin I., Finan P., Johnston D., Mohamdee M., Scott N. (1994). Role of circumferential margin involvement in the local recurrence of rectal cancer. *The Lancet*.

[25] Selby P., Gillis C., Haward R. (1996). Benefits from specialised cancer care. *The Lancet*.

[26] https://languages.oup.com/google-dictionary-en/.

[27] Watts N., Amann M., Arnell N., Ayeb-Karlsson S., Belesova K., Boykoff M. (2019). The 2019 report of the lancet countdown on health and climate change: ensuring that the health of a child born today is not defined by a changing climate. *The Lancet*.

[28] Tennison I., Roschnik S., Ashby B., Boyd R., Hamilton I., Oreszczyn T. (2021). Health care’s response to climate change: a carbon footprint assessment of the NHS in England. *The Lancet Planetary Health*.

[29] Gordon I. O. (2021). Life cycle greenhouse gas emissions of gastrointestinal biopsies in a surgical pathology laboratory. *American Journal of Clinical Pathology*.

[30] https://www.longtermplan.nhs.uk/.

[31] Bhopal A., Norheim O. F. (2023). Fair pathways to net-zero Healthcare. *Nature Medicine*.

[32] Sugrue J., Dagbert F., Park J., Marecik S., Prasad L. M., Chaudhry V. (2017). No clinical benefit from routine histologic examination of stapler doughnuts at low anterior resection for rectal cancer. *Surgery*.

[33] Colorectal cancer statistics (2020). How common is colorectal cancer?. http://www.cancer.org/cancer/colon-rectal-cancer/about/key-statistics.html.

[34] Haq I., Shakeel O., Amjad A., Ullah F., Ali H., Jamal A. (2020). Benefits of outcomes of the microscopic examination of anastomotic donuts after colorectal resection for oncological purposes: a medical record-based study. *Cureus*.

